# The promise and pitfalls: interpreting dual β-lactam success in *Mycobacterium abscessus* with caution

**DOI:** 10.1128/asmcr.00084-25

**Published:** 2025-07-31

**Authors:** Carlos Mejia-Chew

**Affiliations:** 1 Department of Infectious Diseases, The Mater Misericordiae University Hospital, Dublin, Ireland; 2 Department of Infectious Diseases, Washington University School of Medicine, St. Louis, Missouri, USA; Pattern Bioscience, Austin, Texas, USA

## Abstract

*Mycobacterium abscessus* complex (MABSC) poses significant therapeutic challenges and is associated with poor clinical outcomes. There is an urgent need for safer, more effective, and evidence-based treatment options. Dual β-lactam (DBL) therapy has demonstrated promising *in vitro* synergy, and emerging case reports suggest potential benefit as a salvage therapy. Shimamura et al. (ASM Case Rep 1:e00087-24, 2025, https://doi.org/10.1128/asmcr.00087-24) describe two pediatric cases with MABSC pulmonary disease that achieved successful outcomes following the addition of DBL to the treatment regimen. However, the integration of DBL into clinical practice remains challenging due to the lack of standardized synergy testing, limited *in vivo* data, and poorly characterized pharmacodynamics of various DBL combinations at the site of infection. While DBL represents a promising addition to the therapeutic armamentarium for MABSC, its clinical use should be guided by careful appraisal of the currently limited and potentially biased clinical evidence. The paucity of high-certainty data highlights the need for systematic data collection through collaborative multicenter registries and clinical trials to rigorously evaluate the efficacy and safety of DBL regimens.

## COMMENTARY

*Mycobacterium abscessus* complex (MABSC) poses immense therapeutic challenges, often requiring prolonged, toxic, and only partially effective multidrug regimens. The treatment success with current guideline-based therapy is 33%–57%, depending on the MABSC subspecies (1). Current guidelines for the treatment of nontuberculous mycobacterial pulmonary disease recommend using a combination of at least three active antimicrobials for MABSC pulmonary disease (MABSC-PD), guided by *in vitro* drug susceptibility testing (DST) (2, 3). However, for MABSC-PD, only macrolides and amikacin show a positive association between DST and *in vivo* clinical outcomes (1, 4, 5). Moreover, the majority of recommendations in current guidelines are based on non-analytic studies or expert opinion with a very low certainty in estimates of effect (2, 3), highlighting the unmet need for safer, well-tolerated, and evidence-based therapies.

Among the mechanisms of antimicrobial resistance shown by MABSC, there are two that are particularly important for β-lactams. First, MABSC has a thick cell wall, rich in hydrophobic molecules that can limit drug penetration, and two types of enzymes are key for peptidoglycan synthesis: D,D-transpeptidases, the primary target of β-lactams, and L,D-transpeptidases. Second, MABSC possesses a broad-spectrum chromosomal β-lactamase (Bla_Mab_) (6). To devise a guideline-based multidrug regimen, currently, two β-lactams are recommended, given their slower hydrolysis rate by Bla_Mab_: cefoxitin and imipenem (7).

In recent years, there are mounting data showing increased *in vitro* activity and synergy against MBASC with selected dual β-lactams (DBL) therapy and with the addition of a diazabicyclooctanes (DBO) β-lactamase inhibitor (i.e., avibactam, relebactam, nacubactam, zidebactam, and durlobactam) to a β-lactam (8, 9). Scarce *in vivo* animal models support the positive findings (10, 11). However, the promising *in vitro* activity of DBL against MABSC poses its own challenges, as there is no widely available, standardized DST for synergy testing; *in vitro* activity does not always translate to clinical benefit; and the pharmacokinetics and pharmacodynamics of different β-lactams used in combination to achieve synergy at the site of infection remain to be explored.

Due to the limited therapeutic armamentarium for MABSC and traditionally having relied on *in vitro* DST results when composing a multidrug regimen in MABSC, the use of DBL has been gaining traction, particularly with recent case reports showing favorable outcomes (12–15). Recently, Shimamura et al. described two pediatric cases with macrolide-resistant MABSC-PD who received DBL and had clinical and microbiological success. Like previous cases reporting outcomes with the use of DBL for MABSC, clinicians were faced with a lack of alternative therapeutic options, a common scenario in MABSC disease, so DBL was used as salvage therapy to compose and/or maintain an effective multidrug regimen. Such merit is worth highlighting, and when coupled with the established strong *in vitro* activity, it sparks hope in a field long dominated by therapeutic nihilism.

However, the relative contribution of a single intervention in the reported successes is difficult to quantify, as in each case the use of DBL was only one of many therapeutic interventions, including the use of other concurrent and/or sequential antimicrobials (12–14), surgical debridement (15), and bacteriophage therapy (15, 16). Hence, the optimism that accompanies these reports should be tempered with an understanding of the inherent limitations of case-based evidence (17), as the medical literature lacks a meaningful balance of neutral or negative outcomes (18), and the rarity and complexity of *M. abscessus* cases often means that clinicians are motivated to share success stories, especially when novel therapies yield outcomes that deviate from the historically poor prognosis, creating an artificially inflated perception of efficacy. This reflects a well-documented phenomenon of publication bias—the selective reporting of positive results, particularly in observational data (19).

To mitigate the risks of publication bias, journals and clinicians alike must prioritize transparent reporting, including therapeutic failures and adverse outcomes. Clinicians and researchers caring for patients with MABSC disease can also help shed light on the clinical efficacy of DBL for MABSC by engaging in collaborative multicenter registries as a first step to offer more representative data. Other drugs with strong *in vitro* activity against MABS have benefited from such an approach (20, 21). Furthermore, randomized controlled trials (RCTs) remain the gold-standard method to generate evidence of causal associations (22). To overcome the limitations around cost and accrual, adaptive platform trials are being increasingly used in the field of infectious diseases after the COVID-19 pandemic (23), a pathway that may be helpful to determine the best DBL combination among the most promising candidates based on *in vitro* data. Building upon the eagerly anticipated results of the Finding the Optimal Regimen for *Mycobacterium abscessus* Treatment (FORMaT) trial, an adaptive, multi-arm, platform trial designed to find out the best drug combinations to treat MABSC-PD (24), different DBL combinations could be compared using the results of the FORMaT trial as backbone therapy.

Clearly, the advent of DBL to the much-needed and limited treatment options against MABSC represents real progress, but so too are the pitfalls of selective storytelling. So our enthusiasm for dual beta-lactam therapy must remain balanced by a commitment to critical appraisal with cautious interpretation and the development of urgently needed clinical registries and RCTs exploring different DBL combinations for MABSC.
